# Assessment of the Diagnostic Accuracy of the Modified Hodge Test and Modified Carbapenem Inactivation Method for Identifying Carbapenem Resistance Mechanisms in Klebsiella pneumoniae: A Whole Genome Sequencing-Based Exploratory Study

**DOI:** 10.7759/cureus.80312

**Published:** 2025-03-09

**Authors:** Deepika Chakraborty, Mohit Bhatia, Pratima Gupta, Geetha Nagaraj, Varun Shamanna, P. Srinitha, K. V Ashwini, K. L Ravikumar

**Affiliations:** 1 Microbiology, Govind Ballabh Pant Institute of Postgraduate Medical Education and Research, Delhi, IND; 2 Microbiology, All India Institute of Medical Sciences, Rishikesh, IND; 3 Microbiology, Vardhman Mahavir Medical College and Safdarjung Hospital, Delhi, IND; 4 Microbiology, All India Institute of Medical Sciences, Deoghar, IND; 5 Central Research Laboratory, Kempegowda Institute of Medical Sciences, Bengaluru, IND; 6 Biotechnology, Nitte Mahalinga Adyanthaya Memorial Institute of Technology, Udupi, IND; 7 Microbiology, MS Ramaiah University of Applied Sciences, Bengaluru, IND

**Keywords:** carbapenem resistance, klebsiella pneumoniae, modified carbapenem inactivation method, modified hodge test, whole-genome sequencing

## Abstract

Aim: Carbapenem-resistant *Klebsiella pneumoniae* (CRKP) is a major public health concern, particularly in immunocompromised and critically ill patients. Colistin and tigecycline are among the last-resort treatment options, while the primary driver of CRKP emergence is carbapenemase production, especially *Klebsiella pneumoniae* carbapenemase (KPC) and metallo-β-lactamase (MBL). A thorough understanding of its resistance mechanisms is essential for selecting the most effective antimicrobial therapy. This study aimed to evaluate the diagnostic accuracy of the modified Hodge test (MHT) and modified carbapenem inactivation method (mCIM) in detecting molecular resistance mechanisms in CRKP clinical isolates.

Material and methods: This exploratory study consisted of 65 CRKP isolates, which were identified using matrix-assisted laser desorption ionization-time of flight mass spectrometry (MALDI-TOF MS) (Bruker Daltonik GmbH, Bremen, Germany). Antimicrobial susceptibility testing was performed using the BD Phoenix system. The test isolates were subjected to MHT and mCIM and later shipped to the Central Research Laboratory (CRL), Bengaluru, India, where they were subjected to polymerase chain reaction (PCR) and whole-genome sequencing (WGS).

Results: PCR detected *bla_OXA-48-like_*, *bla_NDM-1_*,* bla*_NDM-5_, and* bla_KPC_* genes in 79.7%, 10.2%, 64.4%, and 1.7% CRKP isolates, respectively. The PCR results were concordant with WGS. The MHT demonstrated an overall sensitivity of 60.3%, specificity of 100%, positive predictive value (PPV) of 100%, and negative predictive value (NPV) of 4.2% for detecting carbapenemase production. It showed the highest sensitivity and specificity for *bla_KPC_* (100%) and *bla_OXA-48-like_* (75%) genes, respectively, with the highest PPV for *bla_OXA-48-like_* (91.4%) and NPV for *bla_KPC_* (100%). However, agreement between MHT and PCR for carbapenemase detection was negligible (Kappa: 0.049, p=0.223). A minimal but statistically significant agreement was noted for *bla_OXA-48-like_* detection (Kappa: 0.314, p=0.007), while no significant agreement was observed for *bla_NDM-1_*,* bla_NDM-5_*, or *bla_KPC_* genes. The mCIM had an overall sensitivity of 3.63%, specificity of 100%, PPV of 100%, and NPV of 1.8%. It exhibited the highest sensitivity (4.3%) and specificity (100%) for *bla_OXA-48-like_*_ genes_, with the highest PPV for *bla_OXA-48-like_* (100%), and NPV for *bla_KPC_* (98.1%). No statistically significant agreement was found between mCIM and PCR for carbapenemase detection (Kappa: 0.001, p=0.850).

Conclusions: Comprehensive assessment of the diagnostic accuracy of MHT and mCIM using WGS across a broad spectrum of multi-drug-resistant (MDR) organisms should be conducted at multiple centers to produce reliable data that can guide better clinical management of the patients.

## Introduction

Infections caused by multi-drug resistant (MDR) bacteria are usually treated with last‑resort antibiotics such as carbapenems, polymyxins, or tigecycline [1]. However, with the emergence of carbapenem‑resistant bacteria and the higher toxicity profile of polymyxins and tigecycline, the entire medical fraternity is currently facing a daunting task managing this ongoing menace.

Carbapenem-resistant *Klebsiella pneumoniae* (CRKP) is a member of the family Enterobacteriaceae, which is resistant to the carbapenem group of antibiotics like imipenem, meropenem, and ertapenem. Several mechanisms of carbapenem resistance have been postulated for CRKP, the most common of which is the production of carbapenemase enzymes. There are several genes encoding different carbapenemase enzymes, like *bla_KPC_*, *bla_OXA-48-like_*, and *bla_NDM_*, with the *OXA-48-like* and *NDM* genes most commonly reported from India [2].

The ongoing global transmission of *Klebsiella pneumoniae* carbapenemase (KPC) producing CRKP has had a huge impact on the overall morbidity and mortality in patients contracting infection with this MDR "superbug" [3]. The Centers for Disease Control and Prevention (CDC) have included them under the category of urgent threat, and the WHO has labeled them as Priority 1: critical agents in terms of resistance pattern [4,5]. A previous single-center whole-genome sequencing (WGS)-based study from the Uttarakhand region of India described the presence of *OXA-48-like* and *NDM* genes among CRKP isolates [6]. To the best of our knowledge, there is limited literature on *bla_KPC_-*producing *Klebsiella pneumoniae* in the Uttarakhand region of India.

A consistent testing procedure should be used for the detection of carbapenemase-producing bacteria in light of the alarming rise in their presentation in the clinical isolates. Several phenotypic and genotypic methods for the detection of carbapenemase production are available and have been evaluated globally. One such simple and inexpensive phenotypic test is the modified Hodge test (MHT), which was used extensively before being phased out due to false positive results in extended-spectrum β-lactamase (ESBL)- or AmpC-positive isolates with porin defects, and false negative results in metallo-beta-lactamase (MBL)-producing bacterial isolates, respectively [7]. The Clinical and Laboratory Standards Institute (CLSI) recommended the modified carbapenem inactivation method (mCIM) in 2017. It is a relatively complex phenotypic method and the only organisms in which it can be utilized to detect carbapenemase production are *Pseudomonas aeruginosa* and Enterobacterales [8].

There is a knowledge gap about the reliability of mCIM and MHT in detecting underlying molecular resistance mechanisms among CRKP isolates. The main objective of this single-center exploratory study, conducted at tertiary care teaching hospital in the Uttarakhand region of India, was to evaluate the diagnostic accuracy of MHT and mCIM, using conventional polymerase chain reaction (PCR) as the reference standard [and further verified by WGS], for the detection of carbapenemase genes (*bla_KPC_, bla_OXA-48_, bla_NDM-1_, bla_NDM-5_*) among CRKP test isolates.

## Materials and methods

A time-bound exploratory study (duration: 18 months from February 2021 to July 2022) was conducted at a tertiary care teaching hospital in Uttarakhand, India, after obtaining the approval of the All India Institute of Medical Sciences Ethics Committee (No. AIIMS/IEC/21/73) dated 12/02/2021 and (No. AIIMS/IEC/22/410) dated 05/08/2022.

Inclusion criteria

*Klebsiella pneumoniae* resistant to the carbapenem group of antibiotics (imipenem ± meropenem) isolated from various clinical samples were included in the study. 

Exclusion criteria

Bacteria other than CRKP were excluded from the study.

Clinical samples from inpatients and outpatients of our hospital were processed in the microbiology laboratory following standard guidelines [9], with informed consent obtained beforehand. The bacterial colonies obtained in culture were further identified by colony characteristics, gram staining, catalase test, motility by hanging drop preparation, and matrix-assisted laser desorption ionization-time of flight mass spectrometry (MALDI-TOF MS) (Bruker Daltonik GmbH, Bremen, Germany). The BD Phoenix M50 automated identification/antimicrobial susceptibility testing (ID/AST) system (Becton, Dickinson and Company, Franklin Lakes, New Jersey, USA) was used for performing antibiotic susceptibility testing (ACT), the results of which were interpreted as per the CLSI guidelines 2021 [10]. The *Klebsiella pneumoniae* isolates resistant to imipenem ± meropenem (CRKP) were subjected to MHT and mCIM as per standard protocols [10,11]. Ertapenem was not used for detecting carbapenem resistance due to its unavailability during the study period.

**Figure 1:**
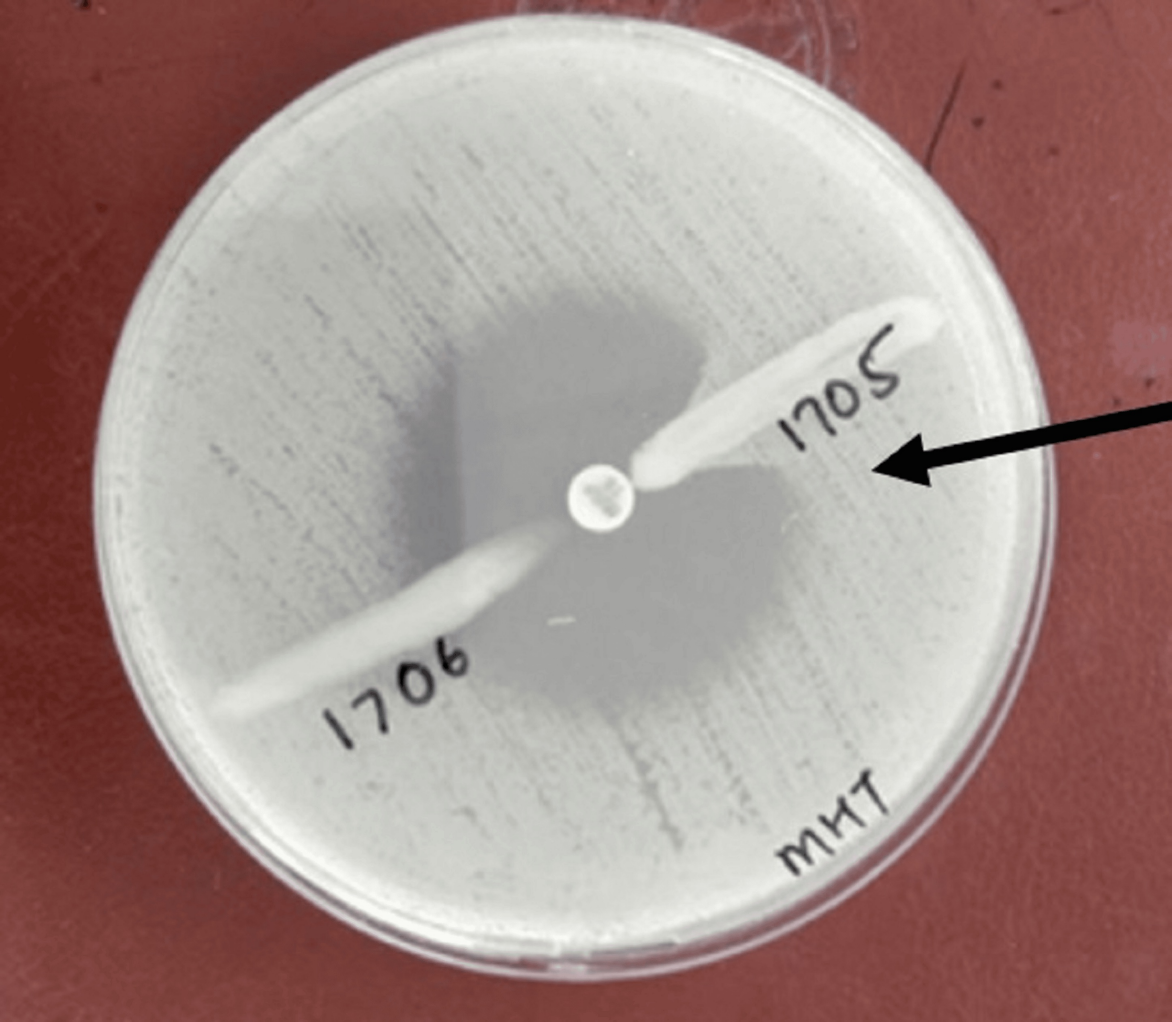
MHT For the positive MHT, *Escherichia coli* ATCC 25922 exhibited a cloverleaf-shaped indentation in the disc diffusion zone (black arrowhead). *Klebsiella pneumoniae* ATCC 1705 and *Klebsiella pneumoniae* ATCC 1706 served as the positive and negative controls, respectively, for the MHT. MHT, modified Hodge test

For MHT, 0.5 McFarland standard suspension of *Escherichia coli *ATCC®25922 (the indicator organism) was prepared in broth or saline. This suspension was then diluted to 1:10 by adding 0.5 mL of the 0.5 McFarland to 4.5 mL saline. A lawn of the 1:10 dilution of *Escherichia coli* ATCC 25922 to a Mueller Hinton agar (MHA) plate was streaked and allowed to dry for 3 to 5 minutes, and a 10 µg meropenem disk was placed in the center of the test area. The test organism was then streaked in a straight line from the edge of the disk to the edge of the plate and incubated overnight at 35±2°C in ambient air for 16-24 hours. The results were interpreted as follows: (a) Enhanced growth: Positive for carbapenemase production; (b) No enhanced growth: Negative for carbapenemase production. Figure 1 illustrates the results of the MHT, with *Klebsiella pneumoniae* ATCC 1705 serving as the positive control and Klebsiella pneumoniae ATCC 1706 as the negative control.

**Figure 2:**
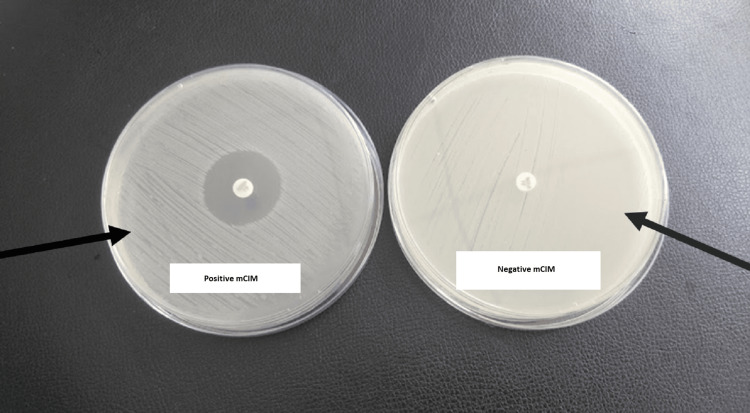
mCIM A positive mCIM is depicted by a zone diameter of 6-15 mm around a 10 µg meropenem disc on a lawn culture of *Escherichia coli* ATCC 25922. A negative mCIM is depicted by no zone of inhibition around a 10 µg meropenem disc on a lawn culture of *Escherichia coli* ATCC 25922. *Klebsiella pneumoniae* ATCC 1705 and *Klebsiella pneumoniae* ATCC 1706 served as the positive and negative controls, respectively, for mCIM. mCIM, modified carbapenem inactivation method

For mCIM, 1 µL loopful of bacteria to be tested were emulsified from an overnight blood agar plate in 2 mL Tryptic Soy Broth (TSB) and vortexed for 10-15 seconds. A 10 µg meropenem disk was added to each tube using sterile forceps ensuring that the entire disk was immersed in the suspension. This was followed by incubation at 35±2°C in ambient air for 4 hours ± 15 minutes. Just before or immediately following the completion of the TSB-meropenem disk suspension incubation, 0.5 McFarland suspension (using the colony suspension method) of *Escherichia coli *ATCC 25922 in nutrient broth or saline was prepared. MHA plate was inoculated with *Escherichia coli * ATCC 25922 after having allowed the plates to dry for 3-10 minutes before adding the meropenem disks. The meropenem disk was removed from each TSB-meropenem disk suspension using a 10 µL loop by carefully dragging and pressing the loop along the inside edge of the tube to expel excess liquid from the disk. It was then placed on the MHA plate, which had been previously inoculated with the meropenem-susceptible *Escherichia coli* ATCC 25922 indicator strain. The interpretation of the mCIM is described in Table 1. Figure 2 presents the mCIM results, with *Klebsiella pneumoniae* ATCC 1705 as the positive control and *Klebsiella pneumoniae* ATCC 1706 as the negative control.

**Table 1:** Interpretation of mCIM results

Result	Zone diameter	Interpretation
Carbapenemase positive	6 to 15 mm or presence of pinpoint colonies within a 16-18 mm zone	If the test isolate produces a carbapenemase, the meropenem in the disk will be hydrolyzed and there will be no inhibition or limited growth inhibition of the meropenem-susceptible *Escherichia coli* ATCC 25922.
Carbapenemase negative	≥19 mm (clear zone)	If the test isolate does not produce carbapenemase, the meropenem in the disk will not be hydrolyzed and will inhibit growth of the meropenem-susceptible *Escherichia coli* ATCC 25922.
Carbapenemase indeterminate	16-18 mm or ≥19 mm and the presence of pinpoint colonies within the zone	The presence or absence of a carbapenemase cannot be confirmed.

All CRKP isolates were shipped to Central Research Laboratory (CRL), Bengaluru, India, where they were subjected to PCR and gel electrophoresis for detection of *bla_OXA-48_*​​​*, bla_NDM-1_, bla_NDM-5_*_,_* and bla_KPC_ genes*. At CRL, genomic DNA was extracted according to the manufacturer’s instructions (Qiagen QIAamp DNA Mini Kit; Qiagen, Venlo, The Netherlands), regardless of the results of the MHT and mCIM. For PCR, eluted DNA was quantified and assessed for purity using a nanodrop. The primers were diluted 1 µg/µL (master stock) to 100 µg/µL (working stock). The master mix was prepared. The composition of the master mix with volume after standardization is enumerated in Table 2. The primer details are provided in Table 3. Master mix (48 µL) was added along with 2 µL of DNA extract in each reaction well. The reaction was run for 35 cycles. The standard setup for each step is mentioned in Table 4. Gel electrophoresis was performed by adding the samples in the pockets of the 1.5% agarose gel along with positive controls and negative controls. A DNA ladder of 100 bp was added in the center, and the test was allowed to run for 45 minutes to 1 hour. The gel containing the sample was examined under chemiluminescence and gel imaging and analysis system.

**Table 2:** Components used in the PCR experiment PCR, polymerase chain reaction; dNTPs, deoxyribonucleotide triphosphates

Components	Amount (µL)
DNA	2.0
Forward primers	1.0
Reverse primers	1.0
dNTPs	1.0
XT5AAB	5.0
Enzyme	0.25
dH_2_O	39.75

**Table 3:** Primers used for the PCR experiment The primers were designed based on a study by Mlynarcik et al. (2016), titled "Primer Evaluation for PCR and its Application for Detection of Carbapenemases in Enterobacteriaceae" (Jundishapur J Microbiol, 9(1):e29314, doi: 10.5812/jjm.29314). PCR, polymerase chain reaction

Gene	Forward/reverse	Primer sequences(43)	Amplicon size (bp)	
				bla_NDM-1_	Forward	3’-TAAAATACCTTGAGCGGGC-5’	439 bp	
Reverse	5’-AAATGGAAACTGGCGACC-3’						
bla_NDM-5_	Forward	3′-AGCACTACTCGGTAAGGCGG-5’	287 bp	
	Reverse	5′-CGCAACACAGCCTGACTTTC-3′		
bla_KPC_	Forward	3’-TGTTGCTGAAGGAGTTGGGC-5’	340 bp	
	Reverse	5’-ACGACGGCATAGTCATTTGC-3’		
bla_OXA-48-__like_	Forward	3’-AACGGGCGAACCAAGCATTTT-5’	585 bp	
	Reverse	5’-TGAGCACTTCTTTTGTGATGGCT-3’		

**Table 4:** PCR protocol PCR, polymerase chain reaction

Cycle step	Temperature	Time	Numbers of cycles
Initial denaturation	94°C	5 min	35
Denaturation	94°C	30 sec	
Annealing	52°C	30 sec	
Extension	72°C	30 sec	
Final extension	72°C	7 min	

WGS of the extracted DNA from test isolates was also performed at CRL. Double-stranded DNA libraries with 450 bp insert sizes were prepared and sequenced using 150 bp paired-end chemistry on the Illumina platform. Spades v3.14 [12] was used to generate contigs, which were annotated with Prokka v1.5 for the genomes passing QC, using the in-house pipelines described in protocols.io (https://www.protocols.io/view/ghru-genomic-surveillance-of-antimicrobial-resista-bp2l6b11kgqe/v4) (ref: https://academic.oup.com/cid/article/73/Supplement_4/S267/6447014) [13]. Multi-locus Sequence Typing (MLST), K- and O-antigen types, virulence factors, and antimicrobial resistance (AMR) genes were identified using the tool described here: (https://www.nature.com/articles/s41467-021-24448-3).

For statistical analysis, categorical variables were presented as proportions while continuous variables were presented as mean with standard deviation (SD). Cohen's kappa coefficient (κ) was calculated for evaluating the agreement between the different testing methods. The sensitivity, specificity, and positive and negative predictive values (NPV) of the phenotypic screening tests for carbapenemase production were also calculated using PCR (and WGS) as the gold standard for detection of *bla_KPC_, bla_OXA-48_, bla_NDM-1_,* and* bla_NDM-5_* genes. All statistical tools were two-tailed and a significant level p of <0.05 was used. Data were entered, cleaned, and coded in an MS Excel (Microsoft, Redmond, WA, USA) spreadsheet. Analysis of data was performed using Statistical Package for the Social Sciences (IBM SPSS Statistics for Windows, IBM Corp., Version 25.0, Armonk, NY).

## Results

Baseline characteristics

Of the 65 *Klebsiella pneumoniae* isolates, 40 (61.54%) were from male patients and 25 (38.46%) from female patients, with a male-to-female ratio of 1.60:1. The mean patient age was 44.05±19.16 years. The most common age group was 31-60 years (42/65, 65%), followed by 15-30 years (9/65, 14%), >60 years (8/65, 12%), and 1-14 years (6/65, 9%).

Among the 65 isolates, 59 (90.77%) were from inpatients and 6 (9.23%) from outpatients. The majority were obtained from pus samples (23/65, 35.38%), followed by urine (22/65, 33.85%), blood (15/65, 23.08%), and drain fluid (5/65, 7.69%).

Medical departments contributed 36 (55%) of the isolates, including General Medicine, Paediatrics, Oncology/Haematology, Emergency, Pulmonology, Urology, Gastroenterology, Community and Family Medicine, and COVID ICUs. Surgical departments accounted for 29 (45%) isolates, covering General Surgery, OBG, Orthopaedics, Burns and Plastic Surgery, Cardiovascular Surgery, ENT, Neurosurgery, Pediatric Surgery, Trauma Surgery, and Surgical Gastroenterology.

Phenotypic and genomic profiles of test isolates

All test isolates were identified as Klebsiella pneumoniae by both MALDI-TOF MS and the BD Phoenix system. It was found that 100%, 91%, 97%, 98%, 97%, 81.5%, and 9% of the test isolates were resistant to penicillins, aminoglycosides, fluoroquinolones, beta-lactam/beta-lactamase inhibitor combinations (BL/BLI), cephalosporins, folate pathway inhibitors, and polymyxins, respectively, as depicted in Table 5.

**Table 5:** Percentage of antibiotic susceptibility patterns for 65 Klebsiella pneumoniae isolates as determined by the BD Phoenix M50 ID/AST system ID/AST, identification/antimicrobial susceptibility testing

Antibiotics	BD Phoenix M50 automated ID/AST system N (%)			
	S	I	SDD	R
Aminoglycosides				
Amikacin	6 (9%)	-	-	59 (91%)
Gentamicin	4 (6%)	-	-	61 (94%)
β-lactam/β-lactamase inhibitors combinations				
Piperacillin-tazobactum	1 (1.5%)	1 (1.5%)	-	63 (97%)
Amoxicillin-clavulanate	1 (1.5%)	1 (1.5%)	-	63 (97%)
Fluoroquinolones				
Ciprofloxacin	2 (3%)	-	-	63 (97%)
Levofloxacin	2 (3%)	-	-	63 (97%)
Cephalosporins				
Ceftazidime	2 (3%)	-	-	63 (97%)
Cefepime	1 (1.5%)	-	1 (1.5%)	63 (97%)
Cefotaxime	2 (3%)	-	-	63 (97%)
Carbapenems				
Imipenem	1 (1.5%)	1 (1.5%)	-	63 (97%)
Meropenem	-	-	-	65 (100%)
Folate pathway inhibitors				
Co-trimoxazole	12 (18%)	-	-	53 (82%)
Tetracyclines				
Tetracycline	10 (15%)	39 (60%)	-	16(25%)
Penicillins				
Ampicillin	-	-	-	65 (100%)
Monobactams				
Aztreonam	3 (5%)	-	-	62 (95%)
Lipopeptides				
Colistin	-	60 (92%)	-	5 (8%)
Phenicols				
Chloramphenicol	6 (9%)	12 (19%)	-	47 (72%)
*S, sensitive; I, intermediate; SDD, susceptible dose-dependent; R, resistant				

**Figure 3:**
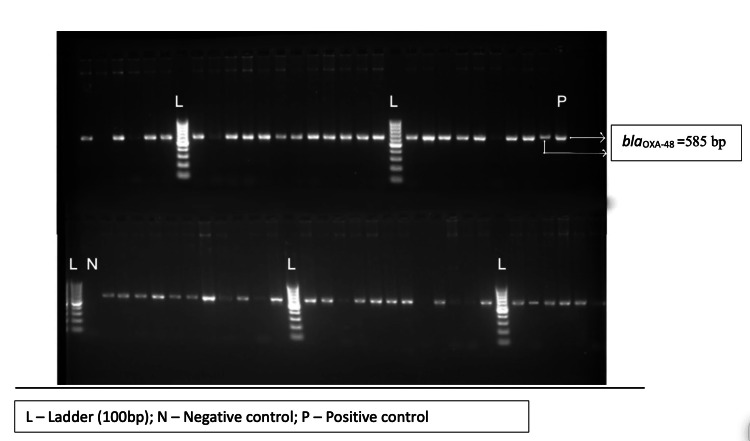
Agarose gel electrophoresis of PCR amplification fragments for detection of blaOXA-48-like gene in test isolates PCR, polymerase chain reaction

**Figure 4:**
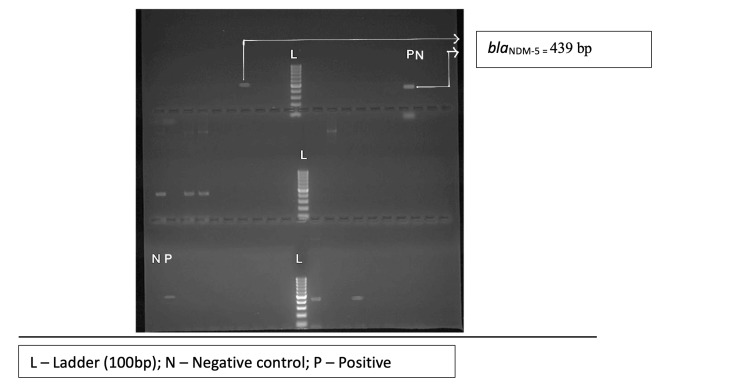
Agarose gel electrophoresis of PCR amplification fragments for detection of blaNDM-1 gene in test isolates PCR, polymerase chain reaction

**Figure 5:**
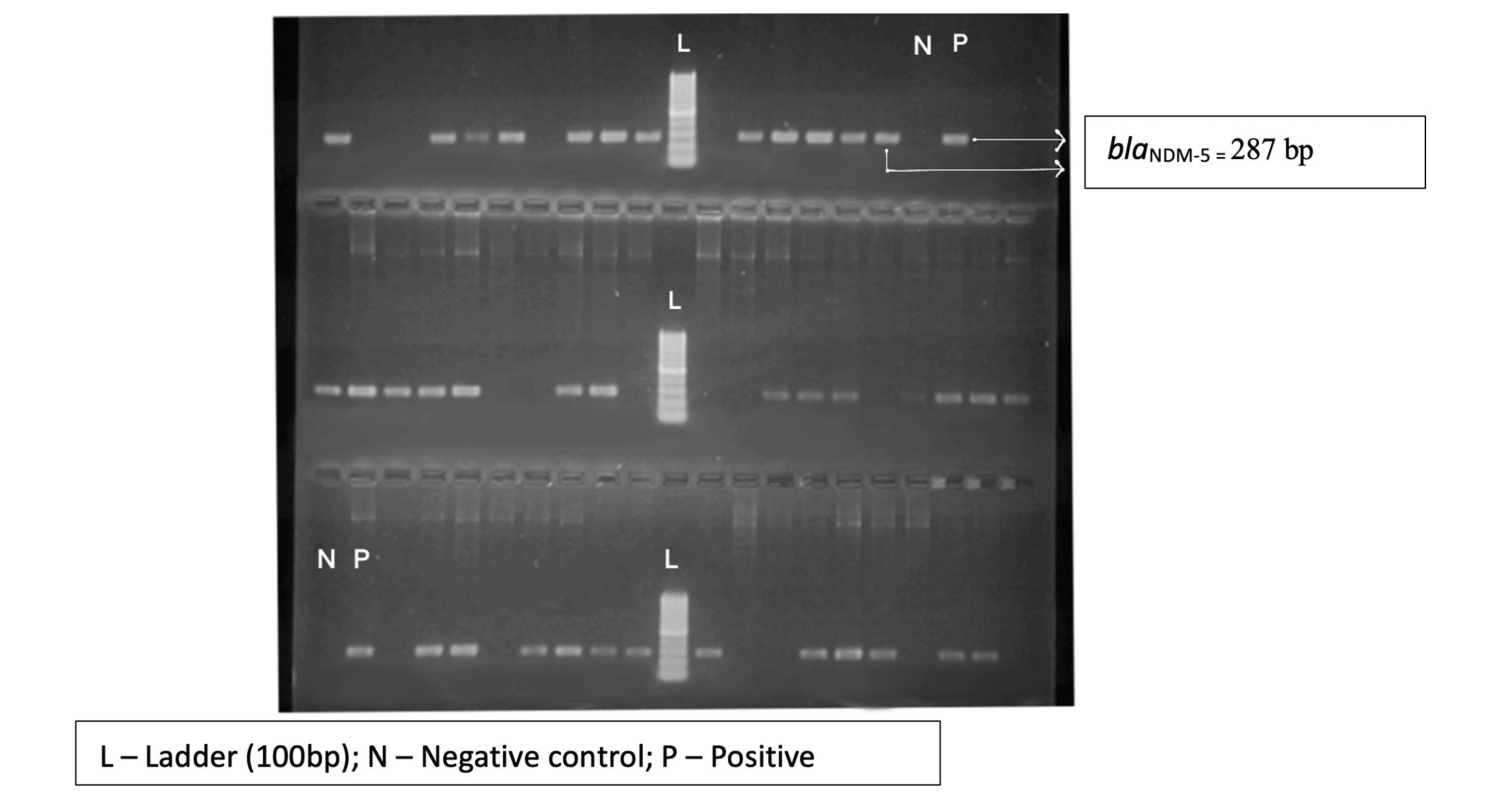
Agarose gel electrophoresis of PCR amplification fragments for detection of blaNDM-5 gene in test isolates PCR, polymerase chain reaction

**Figure 6:**
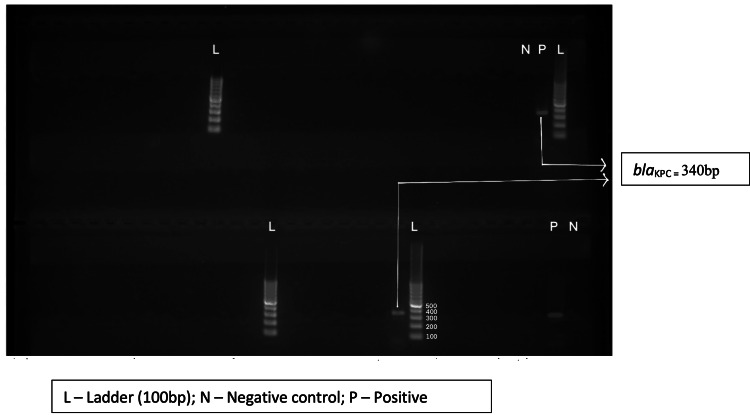
Agarose gel electrophoresis of PCR amplification fragments for detection of blaKPC gene in test isolates PCR, polymerase chain reaction

Out of 65 test isolates, only 59 isolates could be revived and subjected to PCR assays for carbapenemase encoding genes (*bla_KPC_, bla_NDM-1_, bla_NDM-5_*,* and bla_OXA-48-like_*) and the end product was analyzed using gel electrophoresis (Figures 3-6). The *bla_OXA-48-like_, bla_NDM-1_, and bla_NDM-5_*genes were detected in 47 (79.7%), 6 (10.2%), and 38 (64.4%) isolates, respectively. The *bla_KPC_* gene was detected in one isolate only (prevalence: 1.7%). The following combination of genes, i.e., *bla_OXA-48-like_* and *bla_NDM-1_*, and *bla_OXA-48-like_* and *bla_NDM-5_*, were detected in 2 (3.4%) and 32 (54.2%) isolates, respectively.

Out of 59 isolates that were subjected to PCR, WGS results could be obtained for 58 test isolates only. The quality control result of one isolate was not satisfactory and hence was excluded from further evaluation. Among these, 57 were identified as *Klebsiella pneumoniae,* and one isolate was identified as *Klebsiella quasipneumoniae*.

There was no disparity between the carbapenemase gene results of WGS and conventional PCR. 79.3% (46/58) of isolates harbored *bla_OXA-48-like_* genes. Only two variants of the *bla_OXA-48-like_* gene were detected by WGS, namely *bla_OXA-181_* (16/58 (28%)) and *bla_OXA-232_* (30 (52%)). Out of the 58 test isolates, *bla_NDM-1_* and *bla_NDM-5_* were detected in 6 (10.3%) and 37 (63.8%), respectively. Only 1 (1.7%) test isolates carried the *bla_KPC_*gene. One test isolate that could not be subjected to WGS was positive for *bla_OXA-48-like_*, *bla_NDM-1_,* and *bla_NDM-5_* by conventional PCR. A total of 57 out of 58 WGS confirmed test isolates carried one or more carbapenemase genes in addition to another underlying molecular mechanism of resistance to carbapenems, i.e., a combination of ESBL gene and porin loss of function mutation. There was only one isolate in which no carbapenemase genes were detected by WGS. However, this isolate carried *TEM-1D, CTX-M-15, *and* SHV-1* ESBL genes along with disruption of the Ompk35 porin and insertion mutations of TD amino acids (Thr134Asp135 duplication in loop 3 of the porin) at position 115 of the *Ompk36* gene. This isolate was phenotypically sensitive to imipenem and resistant to meropenem.

The genotypic resistance rate for aminoglycosides, fluoroquinolones, and penicillins was 100%. The genotypic resistance rates for folate pathway inhibitors, cephalosporins, chloramphenicol, tetracycline, and polymyxins were 91%, 83%, 52%, 10%, and 7%, respectively. A comparative summary of the phenotypic and genotypic AMR profile of CRKP test isolates is depicted in Table 6.

**Table 6:** A comparative summary of the phenotypic and genotypic antimicrobial resistance profiles of CRKP test isolates For the calculation of concordance between phenotypic and genotypic susceptibility results of tetracycline and chloramphenicol, only phenotypically sensitive or resistant test isolates were considered for evaluation. Test isolates showing phenotypic intermediate AST results were excluded from the analysis. However, no AMR genes were detected in any of these isolates. AST, antibiotic susceptibility testing; AMR, antimicrobial resistance

Antibiotic class	Antibiotic genes	Phenotypic antimicrobial resistance (n (%))	Genotypic antimicrobial resistance (n (%))	Overall concordance (%)
Aminoglycosides (Amikacin, Gentamicin)	aac3, aac6, aadA, aadA2, rmtB, rmtF, armA, aph3, strA, strB,	54 (93.10)	58 (100)	93
Fluoroquinolones (Ciprofloxacin)	qnrB1, qnrB4, qnrS1	56 (96.55)	58 (100)	96.6
Penicillins (Ampicillin)	CTX-M-15, SFO-1	58 (100)	58 (100)	100
Cephalosporins (Ceftriaxone, Cefuroxime axetil, Cefuroxime, Cefepime)	CTX-M-15, SFO-1	56 (96.55)	48 (83)	82.8
Folate pathway inhibitors (Trimethoprim/Sulfamethoxazole)	Sul1, sul2, dfrA1, dfrA12, dfrA14, dfrA30	47 (81.03)	53 (91)	86.3
Polymyxins (Colistin)	pmrB, mgrB	3 (5.17)	4 (7)	88
Tetracycline*	tetA, tetB, tetR, tetD	14 (24.14)	6 (10.34)	22.4
Chloramphenicol*	catA1, catB3, catB4, cmlA5	41 (70.69)	30 (51.72)	41.4

**Figure 7:**
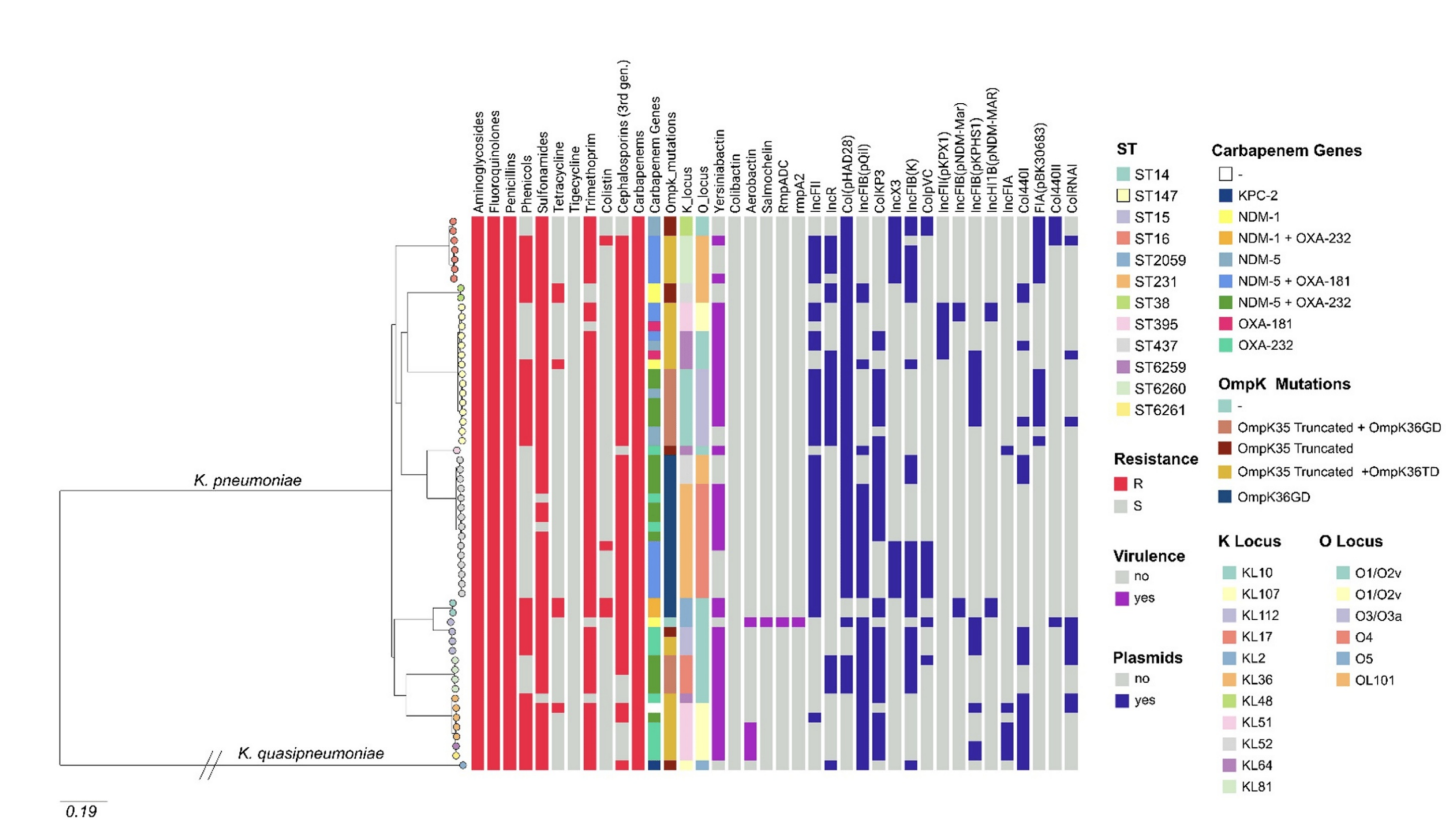
The midpoint-rooted phylogenetic tree obtained by mapping the genomes to the complete genome of Klebsiella pneumoniae strain NZ_CP026011.1 (ST1) using 95,684 filtered SNPs The metadata blocks are arranged to show a comprehensive overview of different resistance patterns to antibiotics, acquired virulence factors, and plasmids in the study. Tree nodes are colored according to their STs. Scale bars represent the number of single-nucleotide polymorphisms per variable site. STs, sequence types; SNP, single nucleotide polymorphism

A summary of the predominant virulence factor genes, plasmid profiles, and predominant AMR genes across sequence types (STs) prevalent in 58 WGS-confirmed test isolates is provided in Table 7. A phylogenetic tree of the test isolates, depicting a comprehensive overview of different antibiotic resistance patterns, acquired virulence factors, and plasmids, is shown in Figure 7 (https://microreact.org/project/k76Lo5vXDfdrXoekSELjaB-deepikautkkpn).

**Table 7:** Summary of predominant virulence factor genes, plasmid profile, and predominant AMR genes across STs prevalent in 58 WGS-confirmed test isolates *STs 14, 15, 38, 231, 395, and 2059 constituted 25% of the CRKP isolates. Six novel STs were found in the present study and were compared to previously reported STs. Four of them were SLV of ST101, one was the SLV of ST231, and another one was the DLV of ST231. The four isolates belonging to ST101-SLV had a mutation in the *phoE* gene, and the allele 601 was assigned to it. The new ST assigned to these isolates was 6260. The ST231-SLV isolate had a mutation in the *mdhB* gene and the new allele number was 462. The new ST assigned to this isolate was 6259. The ST231-DLV had the same mutation in the *mdhB* gene as that of ST231-SLV and an extra mutation in the *tonB* gene, which was assigned an allele number 869. The new ST assigned to this isolate was 6261. ** KL36 (12(20.69%)) and KL51 (9(15.52%)) were dominant K (capsular polysaccharide) loci types in this study. O1, O2, and O4 (lipopolysaccharide surface antigen) serotypes together accounted for 39/58 (67.24%) of CRKP isolates. The presence of OL101 in 10 (17.24%), O3a in 8 (13.79%), and O5 in 1 (1.72%) antigens was also noted. Additionally, O-locus (OL) types displayed ST-specific distribution.  Particularly, O2v1 was found in ST 14, 15, 16, 147, 231, and 395; O2v2 in ST 147 and 231; and O3a in ST 147, respectively. ***The Yersiniabactin iron uptake locus (*ybt*) was identified in 39 (67.2%) of test isolates. In 5 (8.6%) isolates belonging to STs ST15 and ST231, respectively, aerobactin (encoded by iuc gene loci) was found. ****ColKP3 (58.6%), IncFII(K) (50%), IncFIB(pQil) (55.2%), IncFIB(K) (50%), IncR (36.2%), and Col44 0I (32.8%) were the most common plasmid replicons found in the 58 test isolates. STs, sequence types; AMR, antimicrobial resistance; WGS, whole-genome sequencing; SLV, single-locus variants; DLV, double-locus variant; CRKP, carbapenem-resistant *Klebsiella pneumoniae*; ESBL, extended-spectrum β-lactamase

ST*	Test isolates, n (%)	KL-Loci**, n (%)	O-Loci**, n (%)	Predominant virulence factor genes***, n (%)			Predominant plasmids****, n (%)						Predominant AMR genes								
													ESBL genes, n (%)				Carbapenem resistance genes, n (%)				OmpK mutations, n (%)
				Yersiniabactin	Aerobactin	rmpA2	IncFIB(pQil)	IncFIB(K)	Col44 0I	ColKP3	IncFII(K)	IncR	CTXM-15	SHV-28	OXA-1	TEM-1	NDM-1	NDM-5	OXA-181	OXA-232	
14	2 (3.45)	KL2, 2 (100)	O1 & O2v1, 2 (100)	2 (100)	-	-	-	2 (100)	-	2 (100)	2 (100)	-	2 (100)	2 (100)	2 (100)	1 (50)	2 (100)	-	-	2 (100)	2 (100)
15	4 (6.90)	KL2, 1 (25); KL112, 3 (75)	O1 & O2v1,4 (100)	3 (75)	1 (25)	1 (25)	4 (100)	3 (75)	3 (75)	3 (75)	-	-	4 (100)	3 (75)	3 (75)	3 (75)	1 (25)	-	-	3 (75)	3 (75)
16	7 (12.07)	KL48, 2 (28.57); KL81, 5 (71.43)	OL101, 5 (71.43); O1 & O2v1, 2 (28.57)	2 (28.57)	-	-	-	6 (85.71)	-	-	6 (85.71)	4 (57.14)	5 (71.43)	-	1 (14.29)	5 (71.43)	-	7 (100)	5 (71.43)	-	7 (100)
38	2 (3.45)	KL52, 2 (100)	OL101, 2 (100)	-	-	-	2 (100)	2 (100)	2 (100)	-	-	2 (100)	2 (100)	-	2 (100)	2 (100)	2 (100)	-	-	-	2 (100)
147	15 (25.86)	KL51, 3 (20); KL64, 4 (26.67); KL10, 8 (53.33)	O1 & O2v1, 4 (26.67); O2v2, 3 (20); O3a, 8 (53.33)	13 (86.67)	-	-	1 (6.7)	2 (13.33)	2 (13.33)	9 (60)	4 (26.67)	10 (66.67)	15 (100)	15 (100)	8 (53.33)	13 (86.67)	1 (6.67)	12 (80)	5 (33.33)	5 (33.33)	15 (100)
231	5 (8.62)	KL51, 4 (80); KL64, 1 (20)	O1 & O2v2, 4 (80); O1 & O2v1, 1 (20)	5 (100)	2 (40)	-	5 (100)	-	5 (100)	4 (80)	5 (100)	-	2 (40)	2 (40)	-	5 (100)	-	1 (20)	-	4 (80)	5 (100)
395	1 (1.72)	KL64, 1 (100)	O1 & O2v1, 1 (100)	1(100)	-	-	1 (100)	-	-	1 (100)	-	-	-	1 (100)	-	-	-	-	-	1 (100)	1 (100)
437	15 (25.86)	KL36, 12 (80); KL52, 3 (20)	O4, 12 (80); OL101, 3 (20)	7 (46.67)	-	-	12 (80)	9 (60)	3 (20)	9 (60)	9 (60)	-	15 (100)	15 (100)	-	15 (100)	-	13 (86.67)	6 (40)	9 (60)	15 (100)
2059	1 (1.72)	KL107, 1 (100)	O5, 1 (100)	-	-	-	1 (100)	1 (100)	1 (100)	-	1 (100)	1 (100)	-	-	-	-	-	-	-	-	1 (100)
Novel STs	6 (10.34)	KL17, 4 (6.67); KL51, 2 (33.33)	O1 & O2v1, 4 (66.67); O1 & O2v2, 2 (33.33)	6 (100)	2 (33.33)	-	6 (100)	4 (66.67)	3 (50)	6 (100)	2 (50)	4 (6.67)	-	6 (100)	-	2 (33.33)	-	4 (66.67)	-	6 (100)	6 (100)

Agreement analysis of phenotypic tests for detection of carbapenemase production

**Figure 8:**
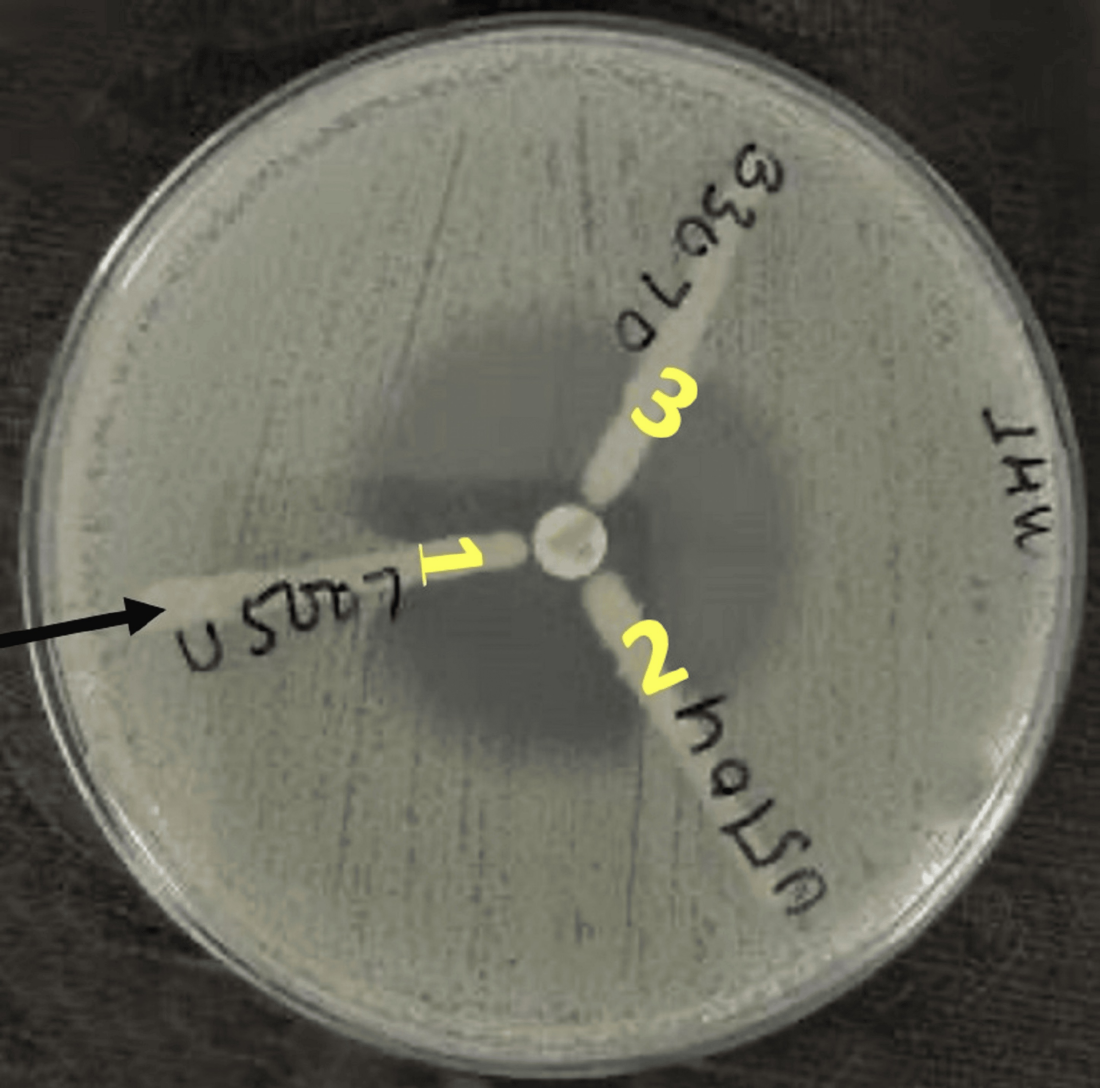
Carbapenemase detection of test isolates by MHT For a positive MHT test, *Escherichia coli *ATCC 25922 developed an indentation like a clover leaf (black arrowhead) in the disc diffusion zone. MHT, modified Hodge test

**Figure 9:**
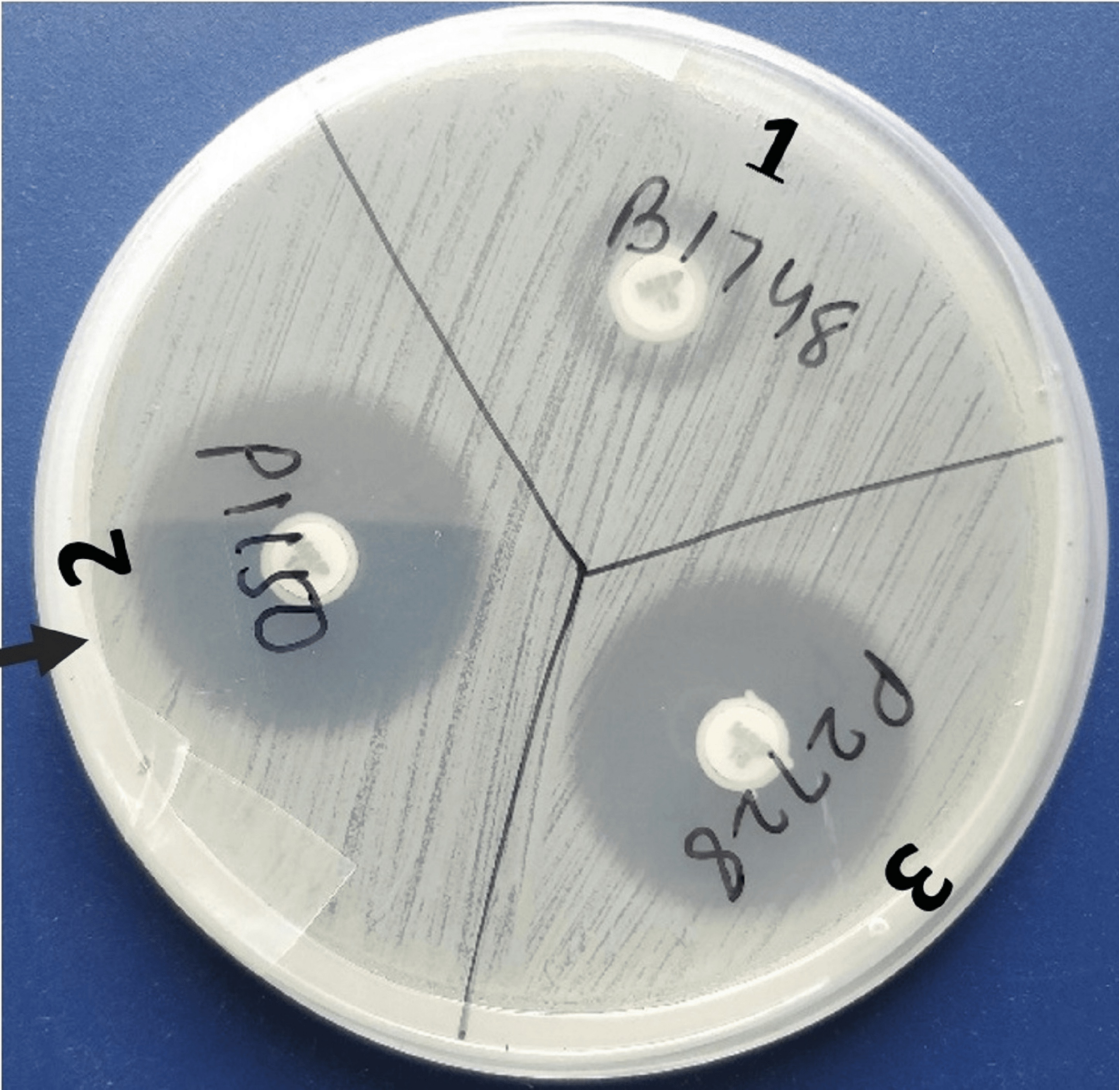
Carbapenemase detection of test isolates by mCIM A positive mCIM is depicted by a zone diameter of 6-15 mm around a 10 µg meropenem disc (black arrowhead) on a lawn culture of *Escherichia coli* ATCC 25922. mCIM, modified carbapenem inactivation method

All 65 test isolates were subjected to both MHT and mCIM, the results of which have been shown in Figure 8 and Figure 9, respectively. A total of 38 (58%) test isolates were tested positive by MHT. Only 2 (3%) were tested positive by mCIM. No statistically significant agreement was observed between the two phenotypic tests (Kappa value: 0.045; 95% CI: -0.17 to 0.111; p-value: 0.233). The results of these two tests have been summarized in Table 8.

**Table 8:** Summary of results of MHT and mCIM mCIM, modified carbapenem inactivation method; MHT, modified Hodge test

Test	mCIM positive	mCIM negative	mCIM indeterminate	Total
MHT positive	2	35	1	38
MHT negative	0	26	1	27
Total	2	61	2	65

Diagnostic accuracy of MHT and mCIM

The overall sensitivity, specificity, positive predictive value (PPV), and NPV of MHT for the detection of carbapenemase production were 60.3%, 100%, 100%, and 4.2%, respectively. MHT had the highest sensitivity and specificity for the detection of *bla_KPC_* (100%) and *bla_OXA-48-like_* (75%) genes, respectively. The PPV and NPV of MHT were highest for the detection of *bla_OXA-48-like_* (91.4%) and *bla_KPC_*(100%) genes, respectively. The overall agreement between MHT and PCR for the detection of carbapenemase genes was negligible and statistically insignificant (Kappa: 0.049; p-value: 0.223; 95% CI: -0.045 to 0.143). While a statistically significant minimal agreement was observed between MHT and PCR for the detection of *bla_OXA-48-like_* (Kappa: 0.314; p-value: 0.007; 95% CI: 0.084 to 0.543), no statistically significant agreement was observed between the two tests for the detection of *bla_NDM-1_*, *bla_NDM-5_*, and *bla_KPC_* genes, respectively. These results are shown in Table 9.

**Table 9:** Diagnostic accuracy and agreement analysis of MHT versus PCR for the detection of carbapenemase genes (blaOXA-48-like, blaNDM-1, blaNDM-5, and blaKPC) The carbapenemase genes (*bla_KPC_, bla_NDM-1_, bla_NDM-5_, and bla_OXA-48-like_*) were detected in two mCIM-positive test isolates. The *bla_OXA-48-like_* were found in two mCIM- and PCR-positive test isolates. The *bla_NDM-5_* genes were found in one mCIM- and PCR-positive test isolate. None of the mCIM-positive isolates were found to be positive for the *bla_KPC_* gene and *bla_NDM-1_*. Interpretation of Cohen’s Kappa (McHugh interrater reliability): 0 to -0.10: No agreement (low negative value); 0 to 0.20: No agreement; 0.21 to 0.39: Minimal agreement, 0.40 to 0.59: Weak agreement; 0.60 to 0.79: Moderate agreement; 0.80 to 0.90: Strong agreement, >0.90: Almost perfect agreement MHT, modified Hodge test; PCR, polymerase chain reaction; PPV, positive predictive value; NPV, negative predictive value

Carbapenemase gene	Sensitivity (%)	Specificity (%)	PPV (%)	NPV (%)	Cohen’s Kappa coefficient	p-value	95% CI	Level of agreement and statistical significance
bla_OXA-48–like_	68.1	75	91.4	62.5	0.314	0.007	0.084 to 0.543	Minimal (statistically significant)
bla_NDM-1_	33.3	37.7	5.7	83.3	-0.092	0.172	-0.237 to 0.053	No agreement (statistically insignificant)
bla_NDM-5_	55.3	33.3	60	70.8	-0.111	0.393	-0.359 to 0.137	No agreement (statistically insignificant)
bla_KPC_	100	41.4	2.9	100	0.023	0.404	-0.022 to 0.068	No agreement (statistically insignificant)
Overall diagnostic accuracy and agreement	60.3	100	100	4.2	0.049	0.223	-0.045 to 0.143	No agreement (statistically insignificant)

The overall sensitivity, specificity, PPV, and NPV of mCIM for detection of carbapenemase production were 3.63%, 100%, 100%, and 1.8%, respectively. mCIM had the highest sensitivity (4.3%) and specificity (100%) for the detection of *bla_OXA-48-like_* genes. The PPV and NPV of mCIM were highest for the detection of *bla_OXA-48-like_* (100%) and *bla_KPC_* (98.1%) genes, respectively. Overall, no statistically significant agreement was observed between mCIM and PCR for the detection of carbapenemase genes (Kappa: 0.001; p-value: 0.850; 95% CI: -0.001 to 0.003). These results are shown in Table 10.

**Table 10:** Diagnostic validity and agreement analysis of mCIM versus PCR for the detection of carbapenemase genes (blaOXA-48-like, blaNDM-1, blaNDM-5, and blaKPC) The carbapenemase genes (*bla_KPC_, bla_NDM-1_, bla_NDM-5_* and *bla_OXA-48-like_*) were detected in 35(59.3%) MHT-positive test isolates. The *bla_OXA-48-ike_* genes were detected in 32 out of 35 MHT- and PCR-positive test isolates. The *bla_KPC_, bla_NDM-1_,* and *bla_NDM-5_* genes were detected in 2, 21, and 1 MHT- and PCR-positive test isolates, respectively. Interpretation of Cohen’s Kappa (McHugh interrater reliability): 0 to -0.10: No agreement (low negative value); 0 to 0.20: No agreement; 0.21 to 0.39: Minimal agreement, 0.40 to 0.59: Weak agreement; 0.60 to 0.79: Moderate agreement; 0.80 to 0.90: Strong agreement, >0.90: Almost perfect agreement PCR, polymerase chain reaction; mCIM, modified carbapenem inactivation method; PPV, positive predictive value; NPV, negative predictive value

Carbapenemase gene	Sensitivity (%)	Specificity (%)	PPV (%)	NPV (%)	Cohen’s Kappa coefficient	p-value	95% CI	Level of agreement and statistical significance
bla_OXA-48–like_	4.3	100	100	20	0.018	0.467	-0.007 to 0.043	No agreement (statistically insignificant)
bla_NDM-1_	0	96.1	0	89.1	-0.054	0.628	-0.112 to 0.005	No agreement (statistically insignificant)
bla_NDM-5_	2.8	95.2	50	36.4	-0.015	0.665	-0.091 to 0.061	No agreement (statistically insignificant)
bla_KPC_	0	96.4	0	98.1	-0.023	0.85	-0.054 to 0.008	No agreement (statistically insignificant)
Overall diagnostic accuracy and agreement	3.63	100	100	1.8	0.001	0.850	-0.001 to 0.003	No agreement (statistically insignificant)

## Discussion

In the present study, there was 100% concordance in test isolate identification results of MALDI-TOF MS and the BD Phoenix system. Similar results were observed in a study done by Kilic et al. between May 2011 and October 2015 [14]. Out of 65 test isolates that were included in the study, 58 were subjected to WGS. Fifty-seven of these isolates were identified as *Klebsiella pneumoniae* and one was identified as *Klebsiella quasipneumoniae* by WGS. A study conducted by Long et al. raised serious concerns about the potential pitfalls in current clinical microbiology laboratory identification techniques as both *Klebsiella pneumoniae* and *Klebsiella quasipneumoniae* have biochemically overlapping phenotypes and are commonly misidentified even with advanced microbial identification systems like MALDI-TOF MS and automated ID/AST machines [15].

In this study, a notable proportion of isolates displayed resistance to several antibiotics like aminoglycosides (91%), fluoroquinolones (97%), BL/BLI (98%), cephalosporins (97%), folate pathway inhibitors (81.5%), and polymyxins (9%). This resistance pattern aligns with similar trends observed in studies by Bhatia et al. and Mahmud et al. among Enterobacterales, including *Klebsiella* spp. [6,16]. Except for penicillin, variable concordance between phenotypic and genotypic data for AMR was seen in our study. The inconsistent genotype-phenotype association may stem from the vast diversity of environmental resistance genes, suggesting a larger reservoir for pathogens to recruit. Common resistance genes in the human microbiome persist even without antibiotic pressure, allowing mobile resistance elements to re-emerge during treatment. The acquisition of novel resistance genes, however, poses a greater threat, as it could lead to resistance against new antibiotics or enhanced mechanisms against existing ones, further limiting treatment options and endangering public health [17].

Our study found no statistically significant agreement between MHT and mCIM results. While no definitive reason can be identified, the limited sample size and lack of external validation may have contributed. Similarly, Gong et al. reported no significant difference between mCIM and MHT in detecting carbapenemase production [18]. However, Farooqui et al. observed a moderate agreement between these two phenotypic tests [19].

In our study, the overall sensitivity and NPV of MHT were higher than mCIM. The specificity and PPV for both MHT and mCIM were 100% each. These results were contrary to the findings of many other similar studies. Zhou et al. reported higher sensitivity, specificity, PPV, and NPV of mCIM over MHT for detecting carbapenemase-producing Enterobacterales (CPE), including CRKP [20]. Gong et al. also reported that for detecting carbapenemase production in Enterobacteriaceae spp., mCIM had higher sensitivity and specificity than MHT [18]. However, Farooqui et al., in their study, observed that mCIM had higher sensitivity but lower specificity than MHT for detecting carbapenemase production [19].

The sensitivity of MHT was highest and lowest for the detection of *bla_KPC_* and *bla_NDM-1_* genes, respectively. The sensitivity of mCIM was highest for the detection of *bla_OXA-48-like_* and lowest for *bla_KPC_* genes. The specificity and PPV of both the phenotypic tests were highest for detecting *bla_OXA-48-like_* genes. However, both MHT and mCIM had the lowest specificity for the detection of *bla_NDM-5_* and the lowest PPV for *bla_KPC_* genes. The NPV of these phenotypic tests was the highest and lowest for detecting *bla_KPC_* and *bla_OXA-48-like_* genes, respectively. Tsai et al. in their study observed that MHT has the highest sensitivity for the detection of *bla_KPC_* (100%), followed by *bla_OXA-48-like_* (95.1%) and *bla_NDM_* (46.7%) genes, respectively, in CPE [21]. Hegde et al. reported that among carbapenem-resistant Enterobacteriaceae (including CRKP), the specificity of MHT was highest and lowest for the detection of *bla_OXA-48-like_* and *bla_NDM-1_* genes, respectively. Another interesting finding in the same study was that the PPV of MHT was highest for the detection of *bla_OXA-48-like_*, followed by *bla_NDM-1_* and *bla_KPC_* genes, respectively [22]. There is a paucity of literature about the diagnostic utility of mCIM in clinical microbiology laboratories. Yu et al. have demonstrated that mCIM has 100% sensitivity and specificity for detecting *bla_KPC-2_*, *bla_NDM-1_,* and *bla_232-type_* carbapenemase genes in Enterobacteriaceae spp. [23]. In a study conducted by Rizvi et al., it was observed that mCIM had 100% sensitivity and specificity for the detection of *bla_OXA-48-like_* genes and 80% sensitivity and 100% specificity for the detection of *bla_NDM-1_* genes [24].

The choice of newer treatment options for hospitalized patients may be significantly influenced by identifying genetic resistance pathways in CRKP isolates. To improve resistome identification, WGS aids in identifying these various underlying mechanisms. Complex mechanisms that are more difficult to anticipate by conventional molecular approaches, such as changes in membrane permeability or up-regulation of efflux pumps, are easily identified by this technique [25]. In the present dataset, *bla_OXA-48-like_* followed by *bla_NDM-5_*and *bla_NDM-1_,*were the most commonly detected genes. The *bla_KPC_*gene was detected in only one test isolate. *bla_OXA-48-like_* is rapidly becoming the most prevalent carbapenemase gene among *Klebsiella pneumoniae* isolates from India, as highlighted by Veeraraghavan et al. and Remya et al. [2,26].

The majority of the WGS-confirmed test isolates (56/58) harbored a carbapenemase-encoding gene along with an ESBL gene and a porin loss-of-function mutation. One isolate harbored carbapenemase encoding genes only. These 57 test isolates were resistant to both imipenem and meropenem. However, there was one isolate in which no carbapenemase-encoding genes were detected. Instead, it was found to be producing ESBL along with disruption of the Ompk35 porin and insertion mutations of TD amino acid at position 115 of the *Ompk36* gene. This isolate was phenotypically susceptible to imipenem but resistant to meropenem. The existence of this unusual phenotype has been primarily explained by the absence of ompK35 and the frameshift mutation in ompK36 in addition to the presence of ESBL genes by Kayama et al. [27].

This study had several limitations, including a small sample size, the exclusion of gram-negative bacterial isolates other than carbapenem-resistant *Klebsiella pneumoniae*, and a random, unstructured selection of test isolates. Additionally, the absence of clinical data prevented any correlation between molecular characterization and treatment outcomes. It is also important to note that this was a single-center study with no external validation from other institutions.

## Conclusions

A multi-center study is essential to assess the accuracy of the MHT and mCIM against WGS across diverse multi-drug-resistant (MDR) organisms. WGS offers a high-resolution approach to detecting resistance mechanisms, providing a precise benchmark for evaluating phenotypic methods’ sensitivity and specificity. Accurate diagnostics are crucial for early detection of carbapenemase-producing organisms (CPOs), enabling optimized antibiotic therapy and improved patient outcomes. If WGS identifies significant shortcomings in MHT or mCIM, alternative rapid diagnostics may be necessary. Reliable testing directly impacts patient care by preventing inappropriate antibiotic use, reducing hospital stays, and lowering mortality rates.

Beyond patient care, WGS enhances infection control and antimicrobial stewardship by tracing MDR transmission routes. It helps identify contamination sources, whether from the environment, medical devices, or patient-to-patient spread, informing targeted prevention strategies. Incorporating WGS into stewardship programs also aids in refining antibiotic use and preserving the efficacy of carbapenems. At the public health level, multi-center WGS data provide real-time epidemiological insights, supporting the early detection of emerging resistance patterns. This knowledge is critical for shaping region-specific antibiotic guidelines and infection control policies. By integrating WGS-based surveillance, hospitals and research institutions can strengthen infection control efforts, refine clinical management, and contribute to the global fight against AMR.
